# Quantifying the Carbon Balance of Forest Restoration and Wildfire under Projected Climate in the Fire-Prone Southwestern US

**DOI:** 10.1371/journal.pone.0169275

**Published:** 2017-01-03

**Authors:** Matthew D. Hurteau

**Affiliations:** Department of Biology, University of New Mexico, Albuquerque, New Mexico, United States of America; Oregon State University, UNITED STATES

## Abstract

Climate projections for the southwestern US suggest a warmer, drier future and have the potential to impact forest carbon (C) sequestration and post-fire C recovery. Restoring forest structure and surface fire regimes initially decreases total ecosystem carbon (TEC), but can stabilize the remaining C by moderating wildfire behavior. Previous research has demonstrated that fire maintained forests can store more C over time than fire suppressed forests in the presence of wildfire. However, because the climate future is uncertain, I sought to determine the efficacy of forest management to moderate fire behavior and its effect on forest C dynamics under current and projected climate. I used the LANDIS-II model to simulate carbon dynamics under early (2010–2019), mid (2050–2059), and late (2090–2099) century climate projections for a ponderosa pine (*Pinus ponderosa*) dominated landscape in northern Arizona. I ran 100-year simulations with two different treatments (control, thin and burn) and a 1 in 50 chance of wildfire occurring. I found that control TEC had a consistent decline throughout the simulation period, regardless of climate. Thin and burn TEC increased following treatment implementation and showed more differentiation than the control in response to climate, with late-century climate having the lowest TEC. Treatment efficacy, as measured by mean fire severity, was not impacted by climate. Fire effects were evident in the cumulative net ecosystem exchange (NEE) for the different treatments. Over the simulation period, 32.8–48.9% of the control landscape was either C neutral or a C source to the atmosphere and greater than 90% of the thin and burn landscape was a moderate C sink. These results suggest that in southwestern ponderosa pine, restoring forest structure and surface fire regimes provides a reasonable hedge against the uncertainty of future climate change for maintaining the forest C sink.

## Introduction

The increasing size and severity of wildfires in the western US poses many ecological and societal challenges, which are likely to be compounded by on-going climate change [1–4]. Efforts to restore forest structure and ecosystem function in dry, fire-prone forests are expanding in scale, but have the potential to create a positive feedback with the climate system as the amount of carbon (C) stored in the forest is reduced [5]. However, the remaining C can be held in a more stable form that is resistant to loss from wildfire because forest restoration treatments are effective at modifying fire behavior and reducing overstory mortality [6–7]. Previous research in southwestern ponderosa pine (*Pinus ponderosa*) forest has demonstrated that a restored condition that is maintained by regular surface fire can store more C than the fire-suppressed condition when the effects of stochastic wildfire are incorporated [8]. However, this result is predicated on reduced tree mortality during wildfire and continued tree growth following wildfire in the restored forest condition.

In the southwestern US, the rate at which forests sequester C is heavily influenced by water availability, which can diminish as temperature increases [9]. Climate projections for the southwestern US include a drier future as higher temperatures increase atmospheric water demand [10]. A warmer, drier future is also projected to increase wildfire probability and size in the western US [11–13]. Yet, fire severity may diminish as post-fire vegetation recovery is influenced by changing climate and less biomass is available to burn [14]. These factors may alter the efficacy of restoration treatments for stabilizing forest C as climate changes.

The ability of forest restoration treatments to moderate wildfire behavior has been demonstrated in many dry forest types [15]. Additionally, reducing tree density through thinning has been shown to reduce drought stress and increase growth and C sequestration relative to a fire-suppressed condition during dry periods [9,16]. These results suggest that management efforts to reduce high-severity wildfire risk and restore surface fire in southwestern ponderosa pine forests could help build the capacity to cope with changing climate.

Given the effects of forest restoration treatments on moderating fire behavior and stabilizing forest C, I sought to determine how the C balance of treatments changes as a function of projected climate. Using climate projections from early- (2010–19), mid- (2050–59), and late-century (2090–99) with a landscape forest simulation model to run 100-year simulations, I hypothesized that 1) treated forests would store more C than untreated forests and that total ecosystem C within treatments would decrease as a function of warming climate because of increased atmospheric water demand and 2) late-century climate would cause the largest decrease in productivity, leading to an overall reduction in wildfire emissions relative to early- and mid-century climate because of reduced biomass available for combustion.

## Materials and Methods

### Study Area

**Fig 1:**
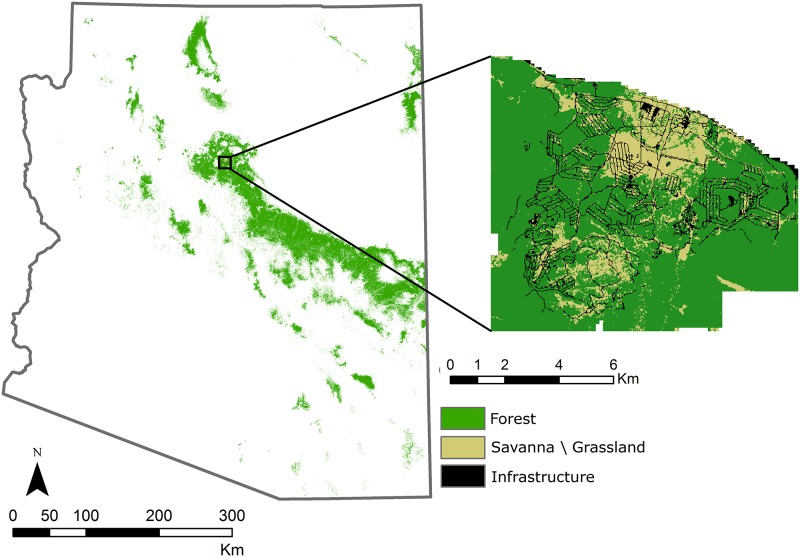
Map of Camp Navajo. The installation is comprised of ponderosa pine forest with some Gambel oak in the understory (dark green) and savanna/grassland (tan) that includes widely spaced ponderosa pine and Gambel oak. The green area within the border of Arizona shows the distribution of ponderosa pine forest within the state. This figure is similar to but not identical to the original image, and is therefore for illustrative purposes only.

Camp Navajo is an 11,610 ha military installation located in northern Arizona, approximately 20 km west of Flagstaff (Fig 1). Mean annual precipitation is 493 mm and is split evenly between summer rains and winter snow. The mean summer maximum temperature is 27°C and the mean winter minimum temperature is -11°C, with a mean annual temperature of 6.9°C (National Climate Data Center, GHCND USC00020678). The forest is predominantly ponderosa pine with a Gambel oak (*Quercus gambelii*) and Rocky Mountain juniper (*Juniperus scopulorum*) component. This landscape was historically shaped by frequent surface fires occurring every 2–20 years [17], but livestock grazing, logging, and fire suppression have altered the forest structure such that tree density, canopy cover, and surface fuels have all increased relative to the fire-maintained condition, transitioning the fire regime from frequent surface fires to infrequent stand-replacing fires [18–19].

### Simulation Model

I used the LANDIS-II forest succession and disturbance model in conjunction with the Century succession, Leaf Biomass Harvest, and Dynamic Fire and Fuels extensions to simulate forest carbon dynamics with different management scenarios and wildfire under projected climate [20–24]. In LANDIS-II, the gridded landscape is populated with tree species represented by biomass in different age classes. Succession and species growth are governed by species-specific life history parameters that control competitive ability, dispersal, and reproduction [20]. Within and among grid cells, cohorts of species grow, compete, disperse, and reproduce. Disturbances can affect cohorts within individual cells or clusters of grid cells. The model requires the landscape be subdivided into abiotically similar ecoregions and that an initial forest communities layer be developed that includes the spatial distribution of age-cohorts of species. Following Hurteau et al. [8] I used the same 150m grid, six ecoregions, and initial communities layer (comprised of ponderosa pine and Gambel oak) that were developed based on soil properties, topographic variables, forest inventory data, and age-size distributions from Fulé et al. [19] and Mast et al. [25] for all simulations.

I used the Century succession extension to simulate pools and fluxes of C, the Leaf Biomass Harvest extension to simulate thinning and prescribed burning treatments, and the Dynamic Fire and Fuels extension to simulate wildfire. The Century succession extension was developed based on the CENTURY soil model [26–28]. The extension simulates above and belowground C and nitrogen pools and fluxes as influenced by species-specific parameters, climate, soils, and their interaction [21–22]. The Century succession extension was parameterized by Hurteau et al. [8] using the SSURGO database (NRCS 2013) and soil samples from the installation. Model spin-up is conducted for the length of time equivalent to the age of the oldest tree cohort and is used to initialize biomass and soil organic matter pools. The maximum cohort age in the initial communties layer at Camp Navajo was 120 years, resulting in a 120 year spin-up period for the model and for this study I used the climate data specific to each climate scenario for spin-up. Soil organic matter decay rates were calibrated following Loudermilk et al. [29] and Martin et al. [30] such that after model spin-up, soil C values fell within the field-sampled range. Species-specific parameter values for the Century succession extension were obtained from the CENTURY user guide, published literature, and US Government databases [9, 27, 31–35] and the distribution of grid cell C values across the landscape compared well with the distribution of empirical values derived from inventory data (see [8]). The only change in Century succession parameterization from Hurteau et al. [8] was a decrease in the probability of establishment from 1 to 0.5 to improve representation of the episodic nature of ponderosa pine regeneration [36]. The probability of establishment parameter is a global parameter that affects all species in the simulation and serves as a maximum value. It limits regeneration only when species-specific parameterizations for growing degree days, drought tolerance, and minimum January temperature are not limiting. This parameter value does not impact Gambel oak re-sprouting following disturbance. I used the Century succession extension to produce annual spatial outputs of net ecosystem exchange (NEE).

Century succession uses monthly averages and standard deviations of minimum and maximum temperature and precipitation to create distributions for drawing monthly climate parameters used during simulations. For this study I used CMIP5 climate data from 41 climate model projections ([37], S1 Table) forced by representative concentration pathway 8.5 (RCP 8.5). RCP 8.5 represents a business-as-usual greenhouse gas emission pathway, with late-century radiative forcing of 8.5 W m^-2^ [38]. I obtained 1/8° bias-corrected constructed-analogs downscaled climate data products from the Downscaled CMIP3 and CMIP5 Climate and Hydrology Projections archive (http://gdo-dcp.ucllnl.org, [37, 39]). From these climate model projections, I developed climate data sets for three periods (2010–2019, 2050–2059, 2090–2099) by calculating the mean daily minimum and maximum temperature and precipitation from the 41 climate projections. I used the mean daily values to calculate the mean and standard deviation of monthly minimum and maximum temperature and precipitation for use in LANDIS-II (S2 Table). Growing season (Apr-Oct) mean minimum and maximum temperature increased by approximately 2°C for each climate period and winter precipitation for all three projected climate periods was greater than for the historic period (S1 Fig).

I used the Leaf Biomass Harvest extension, which is capable of simulating multiple, overlapping treatments, to simulate thinning and prescribed burning [23]. I used the same thinning and prescribed burning treatment developed by Hurteau et al. [8], which removed approximately 30% of the live tree C, targeting the youngest cohorts first, and simulated prescribed burning using a 10-year return interval. The treatments were designed to represent treatments commonly implemented in this forest type to reduce the risk of stand-replacing wildfire. Harvest treatments were implemented on 12% of the installation per year, until all areas designated for treatment were thinned once and prescribed fire treatments were applied to 10% of the landscape per year to simulate a 10-year fire return interval. I excluded the same areas from treatment as Hurteau et al. [8], which included areas with slopes > 14% because they are potential Mexican spotted owl (*Strix occidentalis lucida*) nest sites and include many operational limitations, including the type and timing of treatments (S2 Fig) [40]. I used the Dynamic fire and fuels extension to simulate stochastic wildfire. This extension accounts for changes in fuel characteristics that result from thinning, prescribed burning, and wildfire and links fuel conditions with climate and topographic information to simulate wildfire using a methodology based on the Canadian Forest Fire Behavior Prediction System [41–42]. I used the same wildfire parameterization developed by Hurteau et al. [8], which used fire data from the Coconino National Forest to obtain fire size distribution, ignition frequency, and seasonality. The same fire weather and fuel moisture conditions were used in all climate scenarios to prevent increasing flammability of fuels with mid- and late-century climate, which allowed me to isolate the effects of climate and management on forest C dynamics. Thus, the only interaction between climate scenario and wildfire is through the amount of biomass available to burn. Wildfire simulations used a fire occurrence probability of 2% yr^-1^, which falls in the lowest bin of posterior probabilities for this region estimated by Dickson et al. [43] from empirical fire data. The fuel model parameterization includes an “open” type for when no live trees are present in a grid cell and was parameterized to reflect fire spread consistent with a grassland fire. I used the Dynamic Fire and Fuels extension to produce spatial outputs of annual wildfire severity. Wildfire severity is scaled from 1 to 5, with 5 equal to stand-replacing fire (≥0.9 crown fraction burned (CFB)) and the effects of fire for 1–4 being a function of the age of the cohorts and the fire tolerance of the species. For cohorts other than the youngest (≤10 years), severity classes 1 and 2 represent surface fire, severity class 3 includes some torching of mature trees (0.1≤CFB≤0.495), and severity class 4 includes some high severity patches (0.495≤CFB<0.9). Emissions from wildfire vary as a function of severity class (Table 1).

**Table 1:** Wildfire emissions by severity class. Severity classes 1 and 2 represent surface fire and have the same parameterization. Severity class 3 includes some torching of mature trees. Severity class 4 includes some high severity patches. Severity class 5 is stand-replacing fire.

Severity Class	Mean Emissions (Mg C ha^-1^)	Standard Deviation
1–2	2.27	1.69
3	3.26	4.59
4	7.54	8.32
5	12.88	7.35

### Model Sensitivity

Prior to initiating the simulation experiment, I conducted a series of simulations that excluded management to quantify the effect of projected climate, projected climate and wildfire, and the reduction in the probability of establishment on TEC. I ran simulations with projected early (2010–19), mid (2050–59), and late (2090–99) century climate for comparison with historical (1909–2012) climate simulations from Hurteau et al. [8]. Parameterizations were exactly the same for the projected climate simulations, including a probability of establishment equal to 1.0, to isolate the effect of climate on TEC. I ran 15 replicate simulations for each climate period and each simulation excluded both management and wildfire. I compared simulation year 100 TEC values for the different climate scenarios using ANOVA and Tukey HSD mean comparison. Year 100 TEC under historical climate was significantly greater (p<0.0001) than any of the projected climate scenarios, year 100 TEC under early climate was significantly greater (p<0.002) than both mid- and late-century TEC, and TEC was not significantly different between mid- and late-century climate (S3 Table, S3 Fig). In the Century succession extension, net primary production is influenced by temperature, moisture, nitrogen, and leaf area index. Given that precipitation variability did not differ substantially between historical and projected climate, these results demonstrate that increased temperature under the three different climate periods is sufficient to drive declines in TEC relative to simulations run using historical climate.

To quantify the effect of climate-fire interactions on TEC, I ran the same set of climate scenarios and included wildfire. The wildfire parameterizations, including fire weather, were held constant for all climate scenarios. I compared simulation year 100 TEC values for the different climate scenarios with wildfire using ANOVA and Tukey HSD mean comparison. Year 100 TEC under historical climate was significantly greater (p<0.0001) than any of the projected climate scenarios and there were no significant differences between projected climate scenarios (S4 Table, S4 Fig).

I evaluated the effect of reducing the probability of establishment on total ecosystem carbon (TEC) by running simulations that included wildfire and excluded management for the three climate scenarios used in this study and compared year 100 TEC for scenarios with the two probability of establishment values for each climate scenario. Using a probability of establishment of 0.5 decreased the year 100 TEC by 5.6 to 11.6 Mg C ha^-1^ across climate scenarios. The only statistically significant decrease in TEC occurred for simulations using early (2010–2019) century climate (S5 Table, S5 Fig).

### Simulation Experiment

I ran simulations with two different treatments (control, thin and burn) and three different climates, early-century (2010–19), mid-century (2050–59), and late-century (2090–99) for a total of six different scenarios over a 100-year simulation period with a one-year time-step to quantify the effects of treatment and climate on forest C dynamics. I used climate distributions from the three different periods to determine the effects that a particular climate distribution would have on the ability of the system to sequester and store C. I calculated mean and 95% confidence intervals for total ecosystem carbon (TEC, inclusive of live and dead above and belowground carbon and soil organic carbon) from 15 replicate simulations of each scenario. I used ANOVA and Tukey HSD mean comparison to compare year 100 TEC values among scenarios. To determine the efficacy of treatments in moderating fire severity, I calculated mean fire severity for each scenario using all time-steps from the 15 replicate simulations. I calculated mean fire severity for each grid cell by averaging all fire severity values for any time-step where a fire burned the grid cell for all replicate simulations. I calculated mean and 95% confidence intervals of cumulative fire emissions (wildfire + prescribed fire) for each scenario using the 15 replicates to determine if there were any treatment-climate interactions. I used ANOVA and Tukey HSD mean comparison to compare year 100 cumulative fire emission values among scenarios. To quantify the effects of climate on net ecosystem exchange (NEE) over the simulation period, I calculated mean cumulative NEE by taking the average cumulative NEE of the 15 replicate simulations. NEE is the exchange of carbon between the ecosystem and atmosphere and is simulated from an atmospheric perspective such that negative values indicate the ecosystem is removing C from the atmosphere. The variability between replicate simulations was a function of climate values drawn from the monthly distributions for each time-step and wildfire occurrence and size drawn from the respective distributions for each time-step. I conducted all analyses in R using the Raster package and produced figures using the ggplot2 package [44–46].

## Results

**Fig 2:**
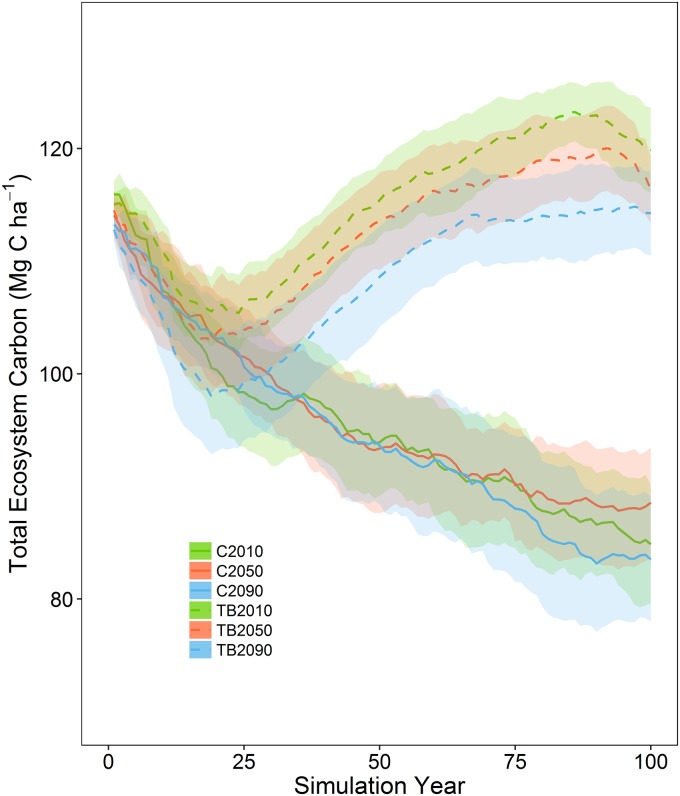
Mean total ecosystem carbon for two simulated treatments (control (C), thin and burn (TB)) under three different climate periods (2010–2019, 2050–2059, 2090–2099) with the same wildfire parameterization over the 100-year simulation period. Shaded areas are the 95% confidence intervals.

Total ecosystem carbon (TEC) showed an initial decline under all scenarios as a result of wildfire (Fig 2). Fire size distributions were fairly consistent between scenarios because wildfire parameterization was held constant between simulations (S6 Fig). Early in the simulation period, late-century climate had the largest effect on the thin and burn TEC, causing the mean to be 3–6% lower than all other scenarios. By simulation year 25, thinning treatments were fully implemented and areas slated for prescribed burning had been burned twice, causing thin and burn TEC to increase as the area affected by high-severity fire decreased because of treatment (Fig 2). Within the thin and burn scenarios, year 100 TEC under early-century climate was significantly greater (*p* = 0.05) than year 100 TEC under late-century climate. All other thin and burn year 100 TEC comparisons were not significantly different. The effects of climate were most pronounced in the thin and burn treatments, with the effects of climate resulting in the highest mean TEC under early-century climate and the lowest mean TEC under late-century climate. In the control simulations, there was little effect of climate on TEC (Fig 2).

**Fig 3:**
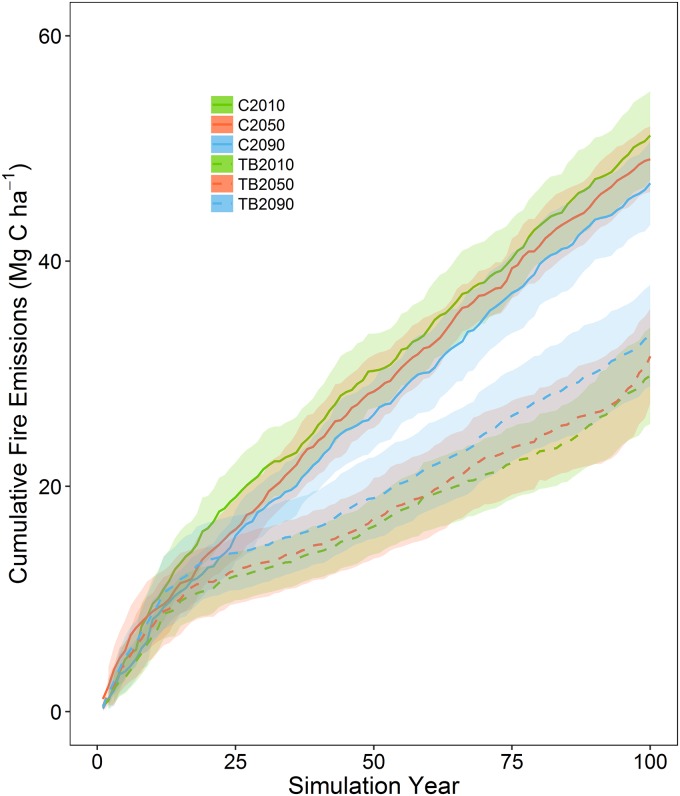
Mean cumulative wildfire and prescribed fire emissions for two simulated treatments (control (C), thin and burn (TB)) under three different climate periods (2010–2019, 2050–2059, 2090–2099) with the same wildfire parameterization over the 100-year simulation period. Shaded areas are the 95% confidence intervals.

Cumulative fire emissions over the 100 year simulation period were highest for the control simulations and lowest for the thin and burn simulations (Fig 3). Year 100 mean cumulative wildfire emissions for the thin and burn were early-century 29.8 Mg C ha^-1^ (sd = 7.7), mid-century 31.5 Mg C ha^-1^ (sd = 7.5), and late-century 33.3 Mg C ha^-1^ (sd = 8.1). Cumulative prescribed fire emissions contributed an additional 6.6–6.8 Mg C ha^-1^ to cumulative fire emission values for the thin and burn. Interestingly, although not significantly different, in the thin and burn scenario late-century climate produced higher cumulative wildfire emissions than the other two climate scenarios. This is in part due to the late-century thin and burn having about 50% more Gambel oak carbon (0.69 Mg C ha^-1^) than the early-century thin and burn (0.45 Mg C ha^-1^, S7 Fig). Although a relatively small contribution to total ecosystem carbon, re-sprouting Gambel oak increases the fuel continuity and smaller individuals are combusted during fire.

**Fig 4:**
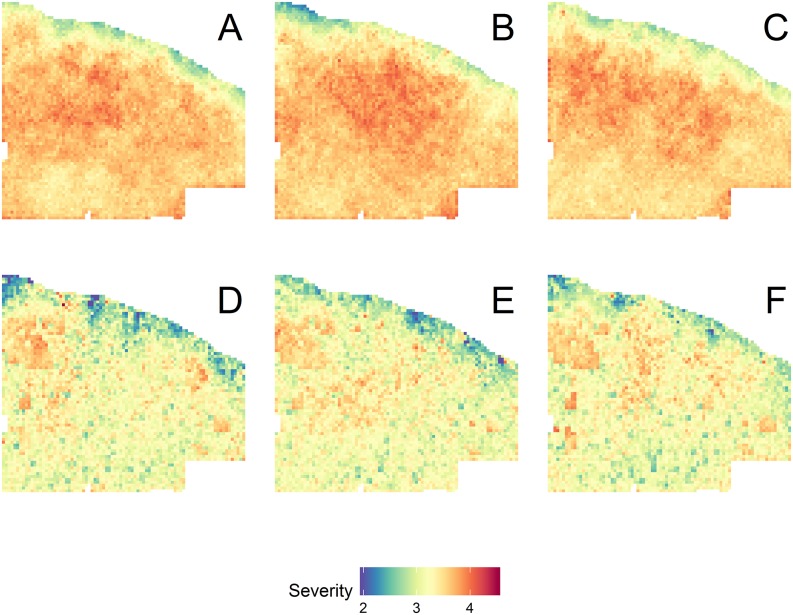
Mean fire severity from 15 replicate simulations for the control 2010–19 climate (A), control 2050–59 climate (B), control 2090–99 climate (C), thin and burn 2010–19 climate (D), thin and burn 2050–59 climate (E), thin and burn 2090–99 climate (F) with the same wildfire parameterization over the 100-year simulation period. The fire severity index ranges from one to five with one being the least and five being the most severe.

**Fig 5:**
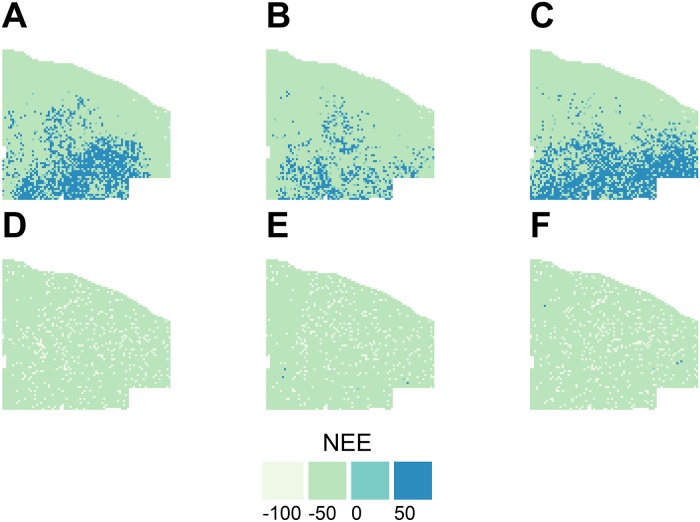
Mean cumulative net ecosystem exchange (NEE, Mg C ha^-1^) from 15 replicate simulations for the control 2010–19 climate (A), control 2050–59 climate (B), control 2090–99 climate (C), thin and burn 2010–19 climate (D), thin and burn 2050–59 climate (E), thin and burn 2090–99 climate (F) with the same wildfire parameterization over the 100-year simulation period. Negative values indicate a sink and positive values a source of carbon to the atmosphere. Bin ranges are -100 (≤-100), -50 (-100< -10), 0 (-10≤10), 50 (>10).

The thin and burn treatment caused a substantial decrease in mean wildfire severity, with little effect of climate scenario (Figs 4 and S8). In the thin and burn scenarios, mean fire severity was generally three or lower, which is indicative of some torching. Whereas in the control scenarios the majority of grid cells had mean fire severity ranging from 3.5–4.2, indicative of torching and some crowning (Fig 4). Fire effects were also evident in the mean cumulative NEE, with control scenarios having 32.8–48.9% of the landscape that was either carbon neutral or a carbon source over the simulation period (Fig 5, Table 2). The majority (>90%) of the landscape under the thin and burn scenarios was a moderate carbon sink (-100 < -10 Mg C ha^-1^) over the simulation period, with 7.2–8.9% of the landscape being a strong carbon sink (≤-100 Mg C ha^-1^, Table 2). Although thinning and prescribed burning shift the age distribution toward older cohorts (S9 Fig), the competitive release can help sustain growth through dry periods [16].

**Table 2:** Percentage of the landscape that had cumulative net ecosystem exchange (NEE, Mg C ha^-1^) that was a strong carbon sink (NEE ≤ -100), a moderate carbon sink (-100<NEE<-10), had little net change in carbon (-10≤NEE≤10), or was a carbon source (NEE>10).

Scenario	NEE≤-100	-100<NEE<-10	-10≤NEE≤10	NEE>10
Control2010	0.5%	56.7%	34.6%	8.2%
Control2050	0.3%	66.9%	31.1%	1.7%
Control2090	0.5%	50.6%	37.1%	11.8%
ThinBurn2010	8.8%	90.9%	0.3%	0%
ThinBurn2050	7.2%	92.0%	0.8%	0%
ThinBurn2090	8.9%	90.1%	1%	0%

**Fig 6:**
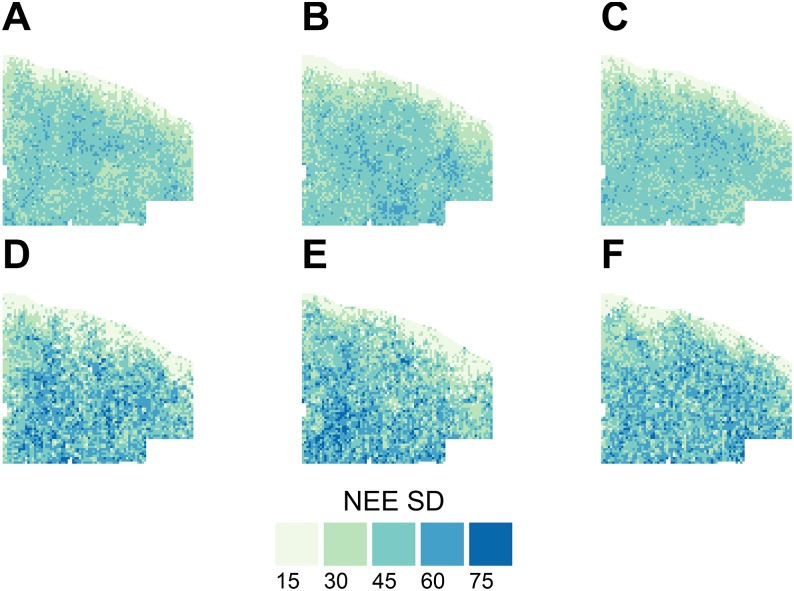
Standard deviation of cumulative net ecosystem exchange (NEE, Mg C ha^-1^) from 15 replicate simulations for the control 2010–19 climate (A), control 2050–59 climate (B), control 2090–99 (C), thin and burn 2010–19 climate (D), thin and burn 2050–59 climate (E), thin and burn 2090–99 climate (F) with the same wildfire parameterization over the 100-year simulation period. Bin ranges are 15 (1–15), 30 (>15–30), 45 (>30–45), 60 (>45–60), 75 (>60–75).

Consistently higher mean fire severity in the control meant a larger fraction of the mature individuals were being killed by fire. The higher rates of mortality translated into more consistent NEE values as demonstrated by the standard deviation of cumulative NEE (Fig 6). Mean fire severity in the thin and burn scenarios was 3 (indicative of some torching of mature individuals) across a large fraction of the landscape. As a result of these fire effects in the thin and burn scenarios, the standard deviation of cumulative NEE was generally larger and had greater variability than in the control scenarios (Fig 6). Thus, the stochastic fire effects drive variability in cumulative NEE.

## Discussion

Projected changes in climate and climate-driven changes in large wildfire frequency present major challenges to conifer forests in the western US [11–12, 47–48] and are likely to exacerbate the current complex of stressors that are already impacting these forests [49]. Fire-exclusion has increased the flammability of dry western conifer forests and restoring natural fire regimes in these systems is central to mitigating the effects of increasingly large disturbance events [49–50]. Yet, the effects of management actions can persist for decades and how these actions and the continued provision of ecosystem services will be affected by changing climate is uncertain [1, 51].

Given the uncertainty about the future global emissions pathway and the role of climate in determining how much carbon a particular system can maintain, I ran simulations using projected climate from three different periods. My results demonstrate that the efficacy of forest restoration treatments that include thinning small diameter trees and restoring surface fires is maintained under projected climate (Fig 2). The restoration of forest structure and the maintenance of that structure with regular surface fire helped sustain the forest C sink, even under increasingly hotter climate (Fig 5). By using climate distributions from three different periods, rather than a continuous trajectory of change, my results demonstrate how forest C dynamics are affected by 100 years of climate that has stabilized at a particular point along the projected trajectory of a high emissions future. These findings suggest that if climate were to stabilize at the late-century conditions simulated in this study, C stability could be maintained with treatments.

Hurteau et al. [8] found that the influence of management on moderating wildfire effects on the system were central to maintaining C stocks and the strength of the C sink under current climate. Similarly, I found that regardless of the climate scenario, management reduced mean fire severity over the simulation period (Figs 4 and S8). This is in part due to the fact that fire is self-limiting and it has been demonstrated that both natural and management fires influence subsequent wildfire size and the area burned by wildfires [52–53]. Interestingly, the effects of wildfire were larger than those of climate on carbon dynamics between the different climate scenarios in the control simulations (Fig 2). This may be a function of simulation length, with the effects of warmer-drier climate requiring additional time to be realized following an initial reduction in TEC resulting primarily from wildfire. Notably, my control simulation TEC results under projected climate show steady decline whereas those of Hurteau et al. [8] increased using the same wildfire parameterization. This difference was driven by forest response to climate as demonstrated by S3 and S4 Figs and a shift in the distribution of fire sizes (S6 and S10 Figs). While the parameterized fire size distribution was held constant between all model runs, when a fire size is drawn from this distribution, it represents the maximum fire size. The simulated fire size is a function of fire weather (also held constant between simulations) and fuels. Under early-century projected climate, mean year 100 Gambel oak C was 2.02 Mg C ha^-1^ (sd = 0.54) and under historical climate mean year 100 Gambel oak C was 0.71 Mg C ha^-1^ (sd = 0.28). The increase in Gambel oak contributed to increased fuel connectivity and enhanced fire spread.

Previous studies have hypothesized the potential for disturbances, such as wildfire, to transition forests to an alternative vegetation type, such as grassland or shrubland, with a marked decrease in the ability of the system to sequester and store C [7, 49]. While my results do not show a complete vegetation type transition with changing climate and wildfire, they do demonstrate the potential for a decline in total ecosystem C and an increase in the proportion of C stored in Gambel oak in the absence of management (Figs 2 and S1). While the Gambel oak results were consistent across climate scenarios for the control, the increase in Gambel oak C under the late-century climate thin and burn scenario suggests a differential response by species to changing climate when wildfire behavior is moderated by treatments (S1 Fig). This result is similar to the findings of Laflower et al. [54] that late-century climate warming and associated drying altered composition in the more species-diverse forests of the Puget Sound.

The impacts of hotter drought on forests present another challenge to sustaining forest C stocks and sequestration in the southwestern US. Periods of high atmospheric water demand, which are likely to become more frequent with warming climate, have produced large-scale mortality events in the past, with a higher frequency of high vapor pressure deficit events projected for the future [48, 55]. Furthermore, evaluation of CMIP5 model projections against historical extreme events show that the model bias for warm extremes is low and for cold extremes is high in the western US [56]. Summer precipitation from monsoon rains is an important precipitation input for this system and monsoon precipitation is biased low and the distribution of precipitation shifts to later in the year over the current distribution [57]. Given the climate model bias and because the modeling framework I used for this study does not include an explicit parameterization for the effects of punctuated, high vapor pressure deficit events, my results carry the caveat that widespread mortality could undermine the sink strength and carbon stock projected under late-century climate. However, the thin and burn scenario is likely to be less impacted by drought than the control because of reduced water competition in the less dense forest, an outcome that has been demonstrated in empirical studies of southwestern ponderosa pine [9, 16, 35].

Another source of uncertainty in my results is the interaction between climate and wildfire. I kept wildfire size distributions and ignition probabilities consistent between simulations to isolate the effects of projected climate and management on C dynamics. As a result, the only pathway through which climate can influence wildfire in the model is through the amount of biomass available to burn. In the southwestern US, the area burned from 2003–2012 increased by 1266% over the 1973–1982 average as a result of increasing spring and summer temperature and earlier spring snowmelt [58]. If this trend of increasing area burned with warming climate holds, as is projected for other regions [11–12], I would expect a steeper decline in TEC than occurred in my control simulations.

The current suite of issues facing forest managers is likely to be compounded by on-going climate change. In forests of the southwestern US, increasingly large wildfires and drought already carry ecological and socioeconomic costs [49, 59], costs that have the potential to rise with changing climate. While managing forests for an uncertain climate future requires a diversity of approaches [60], the results of this study suggest that restoring forest structure and surface fire to southwestern ponderosa pine provides an opportunity to maintain system structure and function, even under the projected warmer, drier future that is likely to have increased fire frequency.

## Supporting Information

S1 Fig Climate data means and standard deviations used in the simulations. Mean monthly minimum and maximum temperature, and mean monthly precipitation for the three projected climate periods (early: 2010–19, mid: 2050–59, late: 2090–99) and the historic period (1909–1912).
(TIFF)

S2 Fig Map of Camp Navajo management areas. The landscape was classified based on fire severity from a series of random ignitions to determine areas with the greatest risk of high-severity fire. Grid cells within the landscape were binned based on mean fire severity. This surface was combined with a slope surface to identify areas for treatment. Areas excluded from management were excluded because slopes >14% limit mechanical harvesting and these areas have the highest likelihood of providing Mexican spotted owl habitat. Areas selected for treatment were ranked based on risk of high-severity fire, with the highest risk areas treated first. The colors represent the management area boundaries as determined from fire severity risk and slope.
(TIF)

S3 Fig Year 100 total ecosystem carbon without wildfire. Comparison of year 100 total ecosystem carbon (TEC) for simulations using projected early (2010–19), mid (2050–59), and late (2090–99) century climate and historical (1909–2013) climate without management or wildfire. With the exception of climate scenario, all parameters were held constant and probability of establishment was equal to 1. Plots constructed from 15 replicate simulations of each scenario.
(TIFF)

S4 Fig Year 100 total ecosystem carbon with wildfire. Comparison of year 100 total ecosystem carbon (TEC) for simulations using projected early (2010–19), mid (2050–59), and late (2090–99) century climate with wildfire and historical (1909–2013) climate with wildfire. With the exception of climate scenario, all parameters were held constant and probability of establishment was equal to 1. Plots constructed from 15 replicate simulations of each scenario.
(TIFF)

S5 Fig Year 100 total ecosystem carbon comparison of seedling establishment probabilities. Comparison of year 100 total ecosystem carbon (TEC) for simulations using projected early (top, 2010–19), mid (middle, 2050–59), and late (bottom, 2090–99) century climate with wildfire for seedling establishment probabilities of 0.5 and 1.0. Plots constructed from 15 replicate simulations of each scenario.
(TIFF)

S6 Fig Distribution of fire sizes. Frequency distributions of fire size constructed from 15 replicate simulations of each scenario. Panels are no management with wildfire in the left column and thinning and burning with wildfire in the right column for early (top, 2010–19), mid (middle, 2050–59), and late (bottom, 2090–99) century climate.
(TIFF)

S7 Fig Mean aboveground carbon for Gambel oak. Mean aboveground carbon for gambel oak (QUGA) for two simulated treatments (control (top), thin and burn (bottom)) under three different climate periods (2010–2019, 2050–2059, 2090–2099) with the same wildfire parameterization over the 100-year simulation period. Shaded areas are the 95% confidence intervals. Note that the y-axis scales differ between plots.
(TIFF)

S8 Fig Area burned by mean fire severity. Histograms of area burned (ha) by fire severity class constructed from 15 replicate simulations of each scenario. Control simulations are in the left column and thin and burn simulations are in the right column for early (top, 2010–19), mid (middle, 2050–59), and late (bottom, 2090–99) century climate.
(TIFF)

S9 Fig Distribution of median tree cohort ages. Distribution of median tree cohort ages in year 100 for the control (top row) and thin and burn scenarios (bottom row) for early (left, 2010–19), mid (middle, 2050–59), and late (right, 2090–99) century climate.
(TIFF)

S10 Fig Distribution of fire sizes under historic climate. Frequency distribution constructed from 15 replicate simulations of the control scenario with wildfire from Hurteau et al. (2016).
(TIFF)

S1 Table CMIP5 climate projections and modeling groups. (PDF)

S2 Table Climate means and standard deviations used in simulations. Monthly mean and standard deviation of minimum and maximum monthly temperature and precipitation used in the LANDIS-II simulations. Values were calculated from CMIP5 climate projections forced using RCP 8.5.
(PDF)

S3 Table Comparison of year 100 total ecosystem carbon without wildfire. Comparison of year 100 total ecosystem carbon for simulations using early (2010–19), mid (2050–59), and late (2090–99) century climate and historical (1903–2013) climate without management or wildfire. Mean separation using Tukey’s HSD and15 replicate simulations of each scenario.
(PDF)

S4 Table Comparison of year 100 total ecosystem carbon with wildfire. Comparison of year 100 total ecosystem carbon for simulations using projected early (2010–19), mid (2050–59), and late (2090–99) century climate with wildfire and historical (1909–2013) climate with wildfire. Mean separation using Tukey’s HSD and 15 replicate simulations of each scenario.
(PDF)

S5 Table Comparison of seedling establishment probabilities. T-test comparison of year 100 total ecosystem carbon for simulations using projected early (2010–19), mid (2050–59), and late (2090–99) century climate with wildfire for seedling establishment probabilities of 0.5 and 1.0.
(PDF)
