# Supplementary figures and images for: Quantifying the Carbon Balance of Forest Restoration and Wildfire under Projected Climate in the Fire-Prone Southwestern US
Source: PLoS One. 2017 Jan 3;12(1):e0169275. doi: 10.1371/journal.pone.0169275 (PMC5207529; doi:10.1371/journal.pone.0169275)

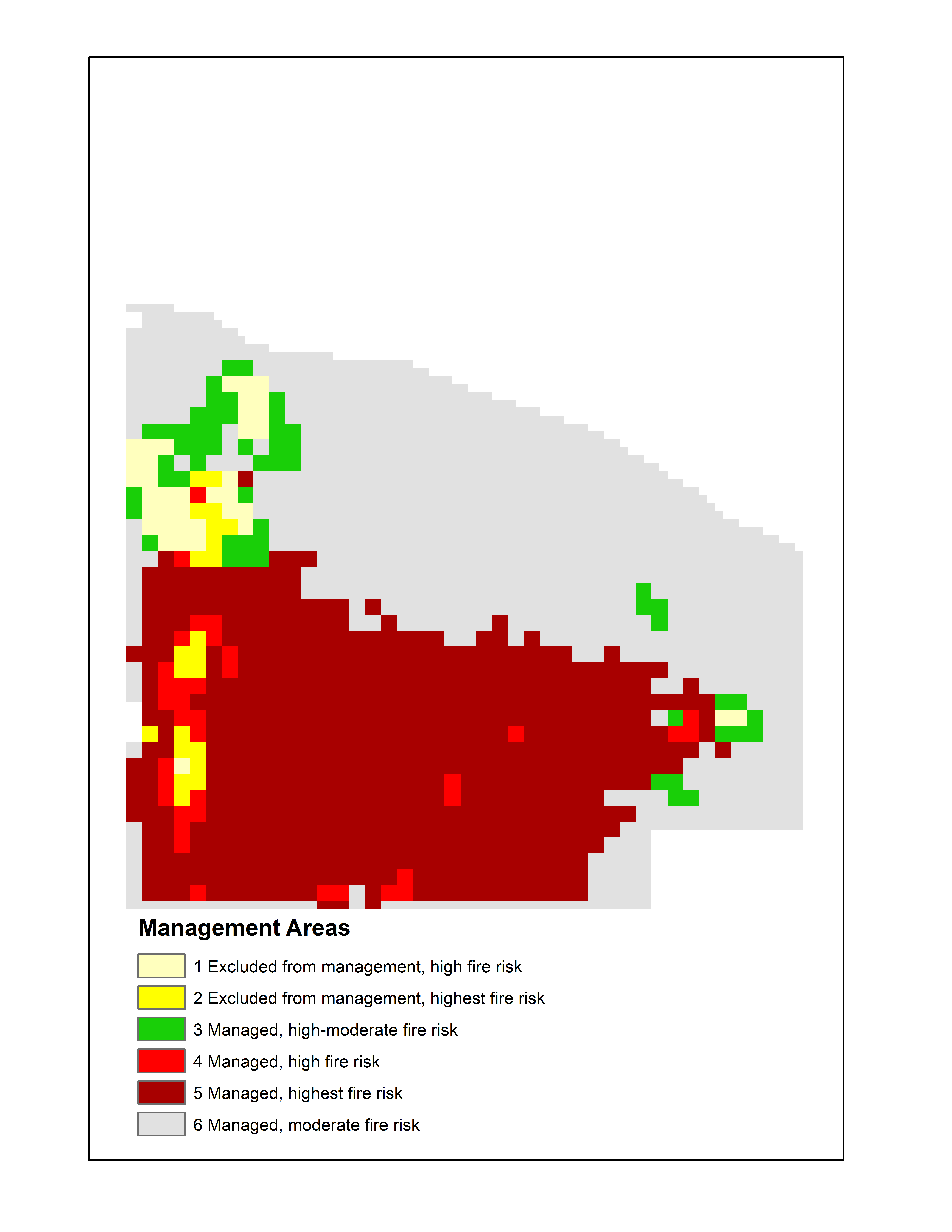

Supplement: S2 Fig — The landscape was classified based on fire severity from a series of random ignitions to determine areas with the greatest risk of high-severity fire. Grid cells within the landscape were binned based on mean fire severity. This surface was combined with a slope surface to identify areas for treatment. Areas excluded from management were excluded because slopes >14% limit mechanical harvesting and these areas have the highest likelihood of providing Mexican spotted owl habitat. Areas selected for treatment were ranked based on risk of high-severity fire, with the highest risk areas treated first. The colors represent the management area boundaries as determined from fire severity risk and slope. (TIF) [file pone.0169275.s002.tif]

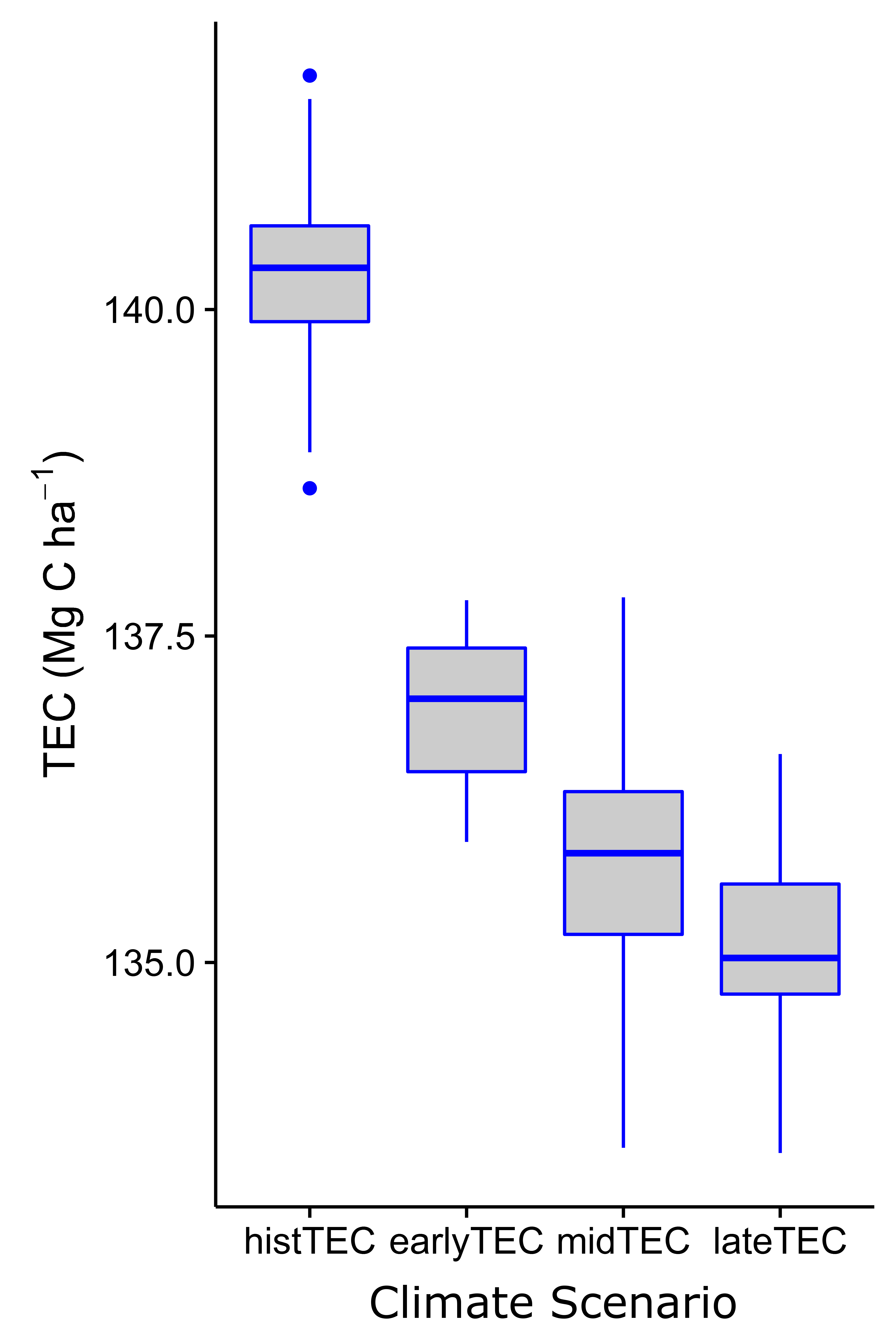

Supplement: S3 Fig — Comparison of year 100 total ecosystem carbon (TEC) for simulations using projected early (2010–19), mid (2050–59), and late (2090–99) century climate and historical (1909–2013) climate without management or wildfire. With the exception of climate scenario, all parameters were held constant and probability of establishment was equal to 1. Plots constructed from 15 replicate simulations of each scenario. (TIFF) [file pone.0169275.s003.tiff]

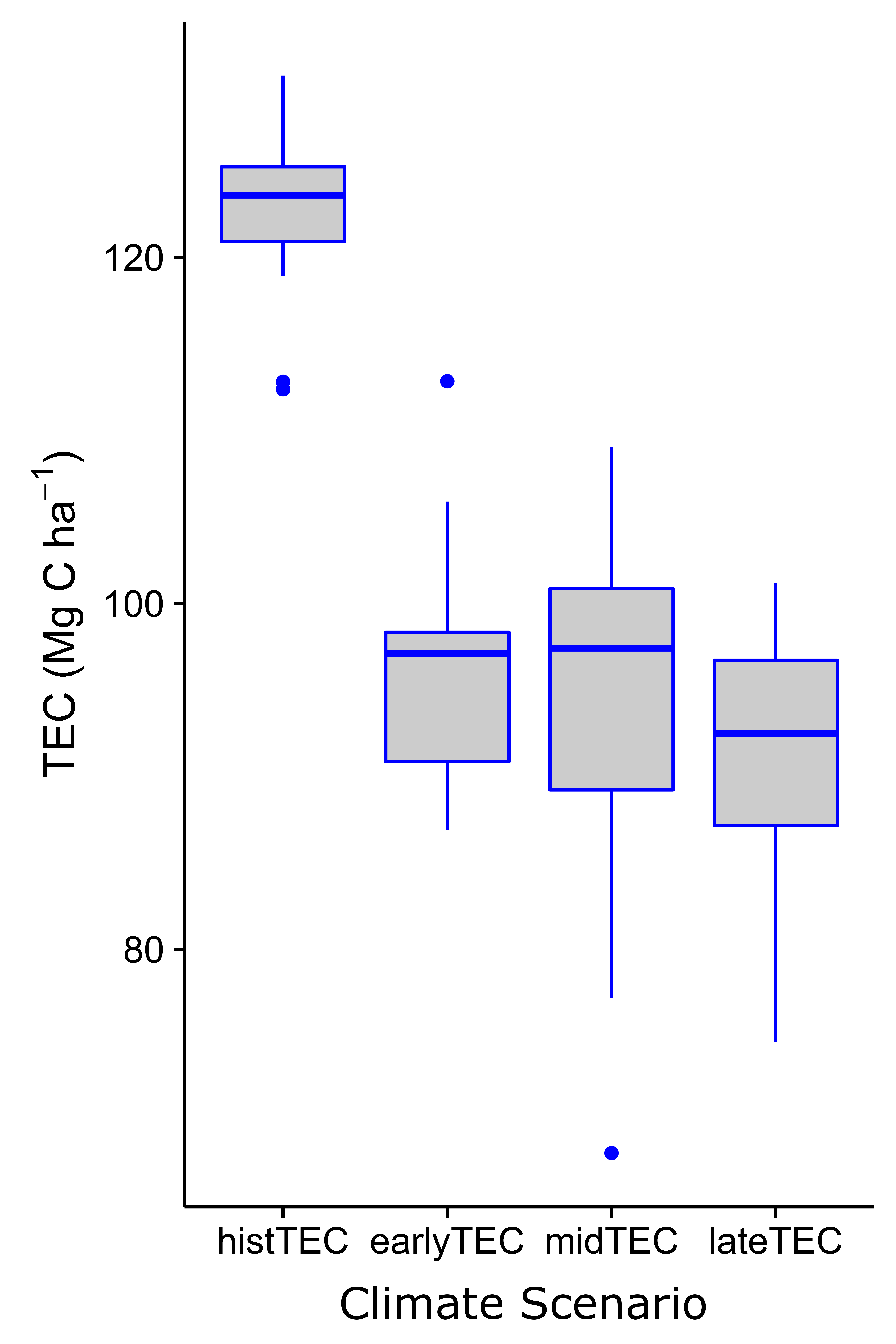

Supplement: S4 Fig — Comparison of year 100 total ecosystem carbon (TEC) for simulations using projected early (2010–19), mid (2050–59), and late (2090–99) century climate with wildfire and historical (1909–2013) climate with wildfire. With the exception of climate scenario, all parameters were held constant and probability of establishment was equal to 1. Plots constructed from 15 replicate simulations of each scenario. (TIFF) [file pone.0169275.s004.tiff]

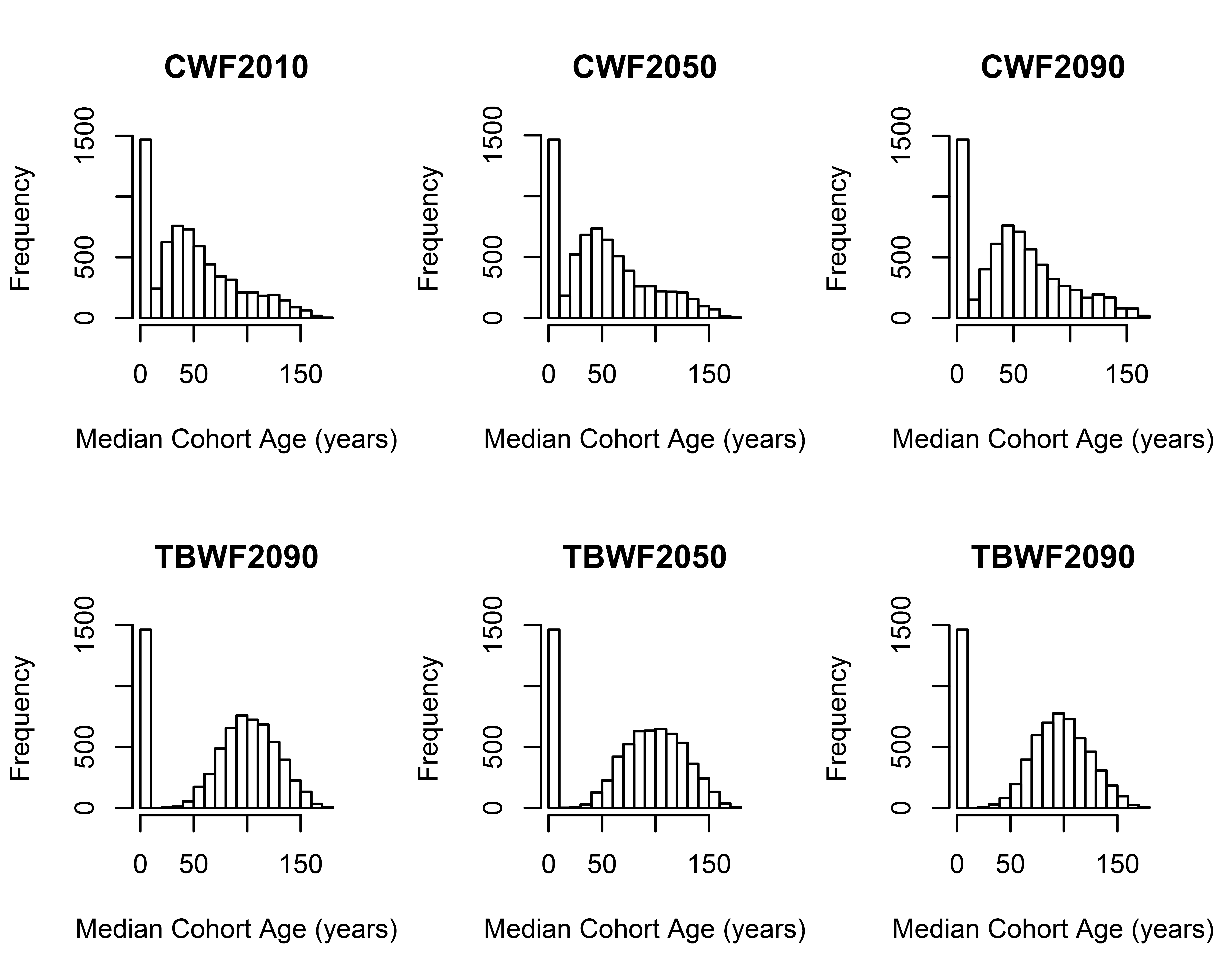

Supplement: S9 Fig — Distribution of median tree cohort ages in year 100 for the control (top row) and thin and burn scenarios (bottom row) for early (left, 2010–19), mid (middle, 2050–59), and late (right, 2090–99) century climate. (TIFF) [file pone.0169275.s009.tiff]

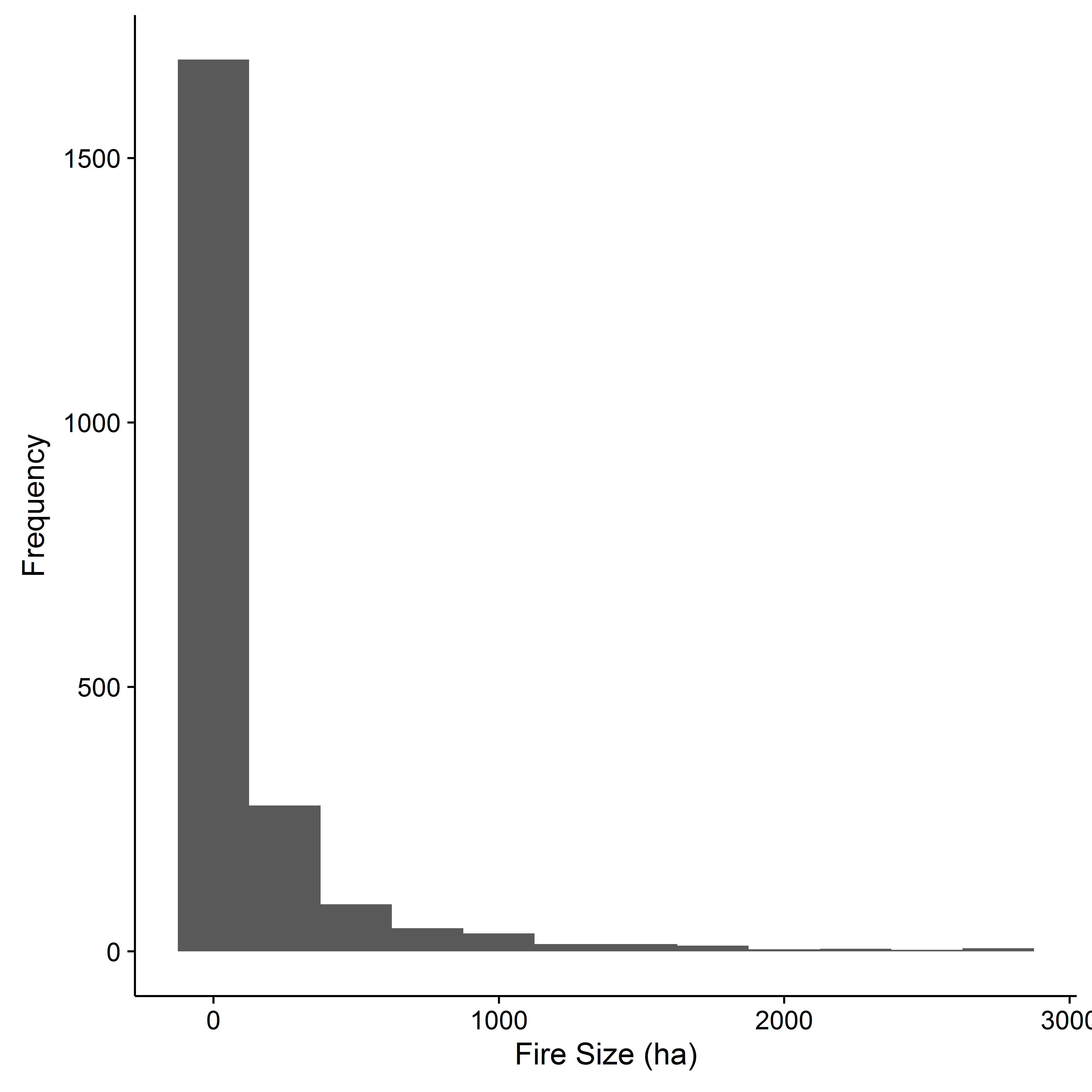

Supplement: S10 Fig — Frequency distribution constructed from 15 replicate simulations of the control scenario with wildfire from Hurteau et al. (2016). (TIFF) [file pone.0169275.s010.tiff]
