# Supplementary material for: Quantifying the Carbon Balance of Forest Restoration and Wildfire under Projected Climate in the Fire-Prone Southwestern US
Source: PLoS One. 2017 Jan 3;12(1):e0169275. doi: 10.1371/journal.pone.0169275 (PMC5207529; doi:10.1371/journal.pone.0169275)
Supplement: S1 Table — (PDF) [file pone.0169275.s011.pdf]

S1 Table: Climate projections were from the following CMIP5 models and CMIP5 modeling groups.

**Modeling Center**	**Institute ID**	**Model Name**	**Simulation**
Commonwealth Scientific and Industrial Research Organization (CSIRO) and Bureau of Meteorology (BOM), Australia	CSIRO-BOM	ACCESS 1.0	access1-0.1.rcp85
Beijing Climate Center, China Meteorological Administration	BCC	BCC-CSM1.1	bcc-csm1-1.1.rcp85
Canadian Centre for Climate Modeling and Analysis	CCCMA	CanESM2	canesm2.1.rcp85<br>canesm2.2.rcp85<br>canesm2.3.rcp85<br>canesm2.4.rcp85<br>canesm2.5.rcp85
National Center for Atmospheric Research	NCAR	CCSM4	ccsm4.1.rcp85<br>ccsm4.2.rcp85
Community Earth System Model Contributors	NSF-DOE-NCAR	CESM1(BGC)	cesm1-bgc.1.rcp85
Centre National de Recherches Météorologiques / Centre Européen de Recherche et Formation Avancée en Calcul Scientifique	CNRM-CERFACS	CNRM-CM5	cnrm-cm5.1.rcp85
Commonwealth Scientific and Industrial Research Organization in collaboration with Queensland Climate Change Centre of Excellence	CSIRO-QCCCE	CSIRO-Mk3.6.0	csiro-mk3-6-0.1.rcp85<br>csiro-mk3-6-0.2.rcp85<br>csiro-mk3-6-0.3.rcp85<br>csiro-mk3-6-0.4.rcp85<br>csiro-mk3-6-0.5.rcp85<br>csiro-mk3-6-0.6.rcp85<br>csiro-mk3-6-0.7.rcp85<br>csiro-mk3-6-0.8.rcp85<br>csiro-mk3-6-0.9.rcp85<br>csiro-mk3-6-0.10.rcp85
NOAA Geophysical Fluid Dynamics Laboratory	NOAA GFDL	GFDL-CM3.1<br>GFDL-ESM2G<br>GFDL-ESM2M	gfdl-cm3.1.rcp85<br>gfdl-esm2g.1.rcp85<br>gfdl-esm2m.1.rcp85
Institute for Numerical Mathematics	INM	INM-CM4	inmcm4.1.rcp85
Institut Pierr-Simon Laplace	IPSL	IPSL-CM5A-LR<br>IPSL-CM5A-MR	ipsl-cm5a-lr.1.rcp85<br>ipsl-cm5a-lr.2.rcp85<br>ipsl-cm5a-lr.3.rcp85<br>ipsl-cm5a-lr.4.rcp85<br>ipsl-cm5a-mr.1.rcp85
Japan Agency for Marine-Earth Science and Technology, Atmosphere and Ocean Research Institute (The University of Tokyo), and National Institute for Environmental Studies	MIROC	MIROC-ESM<br>MIROC-ESM-CHEM	miroc-esm.1.rcp85<br>miroc-esm-chem.1.rcp8.5
Atmosphere and Ocean Research Institute (The University of Tokyo),	MIROC	MIROC5	miroc5.1.rcp85<br>miroc5.2.rcp85

and National Institute for Environmental Studies, and Japan Agency for Marine-Earth Science and Technology			miroc5.3.rcp85
Max-Planck-Institut fur Meteorologie (Max Planck Institute for Meteorology)	MPI-M	MPI-ESM-LR<br>MPI-ESM-MR	mpi-esm-lr.1.rcp85<br>mpi-esm-lr.2.rcp85<br>mpi-esm-lr.3.rcp85<br>mpi-esm-mr.1.rcp85
Meteorological Research Institute	MRI	MRI-CGCM3	mri-cgcm3.1.rcp85
Norwegian Climate Centre	NCC	NorESM1-M	noresm1-m.1.rcp85
