# Supplementary material for: Quantifying the Carbon Balance of Forest Restoration and Wildfire under Projected Climate in the Fire-Prone Southwestern US
Source: PLoS One. 2017 Jan 3;12(1):e0169275. doi: 10.1371/journal.pone.0169275 (PMC5207529; doi:10.1371/journal.pone.0169275)
Supplement: S2 Table — Monthly mean and standard deviation of minimum and maximum monthly temperature and precipitation used in the LANDIS-II simulations. Values were calculated from CMIP5 climate projections forced using RCP 8.5. (PDF) [file pone.0169275.s012.pdf]

S2 Table: Monthly mean and standard deviation of minimum and maximum temperature and precipitation used in LANDIS-II simulations, calculated from CMIP5 climate projections forced using RCP 8.5.

Period	Month	AvgMinT (°C)	AvgMaxT (°C)	StdevT	AvgPpt (mm)	Stdev Ppt
2010-19	1	-7.9	7.7	8.0	7.2	1.1
2010-19	2	-6.9	9.0	8.2	7.0	0.8
2010-19	3	-4.9	11.6	8.5	6.3	0.6
2010-19	4	-1.8	16.5	9.4	3.5	0.7
2010-19	5	1.8	21.7	10.2	1.5	0.2
2010-19	6	6.2	27.6	11.0	1.2	0.2
2010-19	7	11.0	29.3	9.4	6.4	0.7
2010-19	8	10.6	27.8	8.8	7.9	0.7
2010-19	9	6.7	25.1	9.5	5.1	0.7
2010-19	10	0.7	19.8	9.8	4.0	0.5
2010-19	11	-4.5	12.9	8.9	4.8	0.4
2010-19	12	-7.9	8.3	8.3	6.1	0.7
2050-59	1	-6.2	9.4	8.0	7.5	0.6
2050-59	2	-5.1	11.0	8.3	6.9	0.8
2050-59	3	-3.2	13.8	8.7	5.9	0.3
2050-59	4	-0.1	18.7	9.7	2.9	0.4
2050-59	5	3.7	24.0	10.4	1.4	0.3
2050-59	6	8.3	29.9	11.1	1.2	0.2
2050-59	7	13.4	31.3	9.2	6.7	0.4
2050-59	8	12.9	29.8	8.6	8.6	0.9
2050-59	9	8.8	27.3	9.5	5.4	0.6
2050-59	10	2.9	22.3	10.0	4.3	0.6
2050-59	11	-2.9	15.1	9.2	4.2	0.8
2050-59	12	-6.1	10.4	8.4	5.8	0.8
2090-99	1	-3.9	11.7	8.0	8.5	0.9
2090-99	2	-2.9	13.1	8.3	8.2	0.8
2090-99	3	-1.2	15.8	8.7	6.5	1.0
2090-99	4	2.1	21.1	9.7	2.4	0.3
2090-99	5	6.1	26.4	10.4	1.1	0.1
2090-99	6	11.2	32.7	11.0	1.1	0.2
2090-99	7	16.5	34.3	9.2	6.6	0.4
2090-99	8	15.9	32.6	8.6	8.8	0.6
2090-99	9	11.8	30.3	9.5	5.6	0.5
2090-99	10	5.8	25.2	9.9	4.1	0.5
2090-99	11	-0.1	17.9	9.2	4.2	0.6
2090-99	12	-3.9	12.6	8.4	5.6	0.6
