# Supplementary material for: Quantifying the Carbon Balance of Forest Restoration and Wildfire under Projected Climate in the Fire-Prone Southwestern US
Source: PLoS One. 2017 Jan 3;12(1):e0169275. doi: 10.1371/journal.pone.0169275 (PMC5207529; doi:10.1371/journal.pone.0169275)
Supplement: S3 Table — Comparison of year 100 total ecosystem carbon for simulations using early (2010–19), mid (2050–59), and late (2090–99) century climate and historical (1903–2013) climate without management or wildfire. Mean separation using Tukey’s HSD and15 replicate simulations of each scenario. (PDF) [file pone.0169275.s013.pdf]

S3 Table: Comparison of year 100 total ecosystem carbon for simulations using early (2010-19), mid (2050-59), and late (2090-99) century climate and historical (1903-2013) climate without management or wildfire. Mean separation using Tukey's HSD and 15 replicate simulations of each scenario.

Climate Scenario	Difference	Lower 95% CI	Upper 95% CI	P adj
Historic-Early	3.2601236	2.4235886	4.0966589	<0.0001
Late-Early	-1.7401411	-2.5766764	-0.9036058	<0.0001
Mid-Early	-1.2023396	-2.0388749	-0.3658043	0.0019474
Late-Historic	-5.0002647	-5.8368000	-4.1637294	<0.0001
Mid-Historic	-4.4624632	-5.2989985	-3.6259279	<0.0001
Mid-Late	0.5378015	-0.2987338	1.3743368	0.3321615
