# Supplementary material for: Quantifying the Carbon Balance of Forest Restoration and Wildfire under Projected Climate in the Fire-Prone Southwestern US
Source: PLoS One. 2017 Jan 3;12(1):e0169275. doi: 10.1371/journal.pone.0169275 (PMC5207529; doi:10.1371/journal.pone.0169275)
Supplement: S4 Table — Comparison of year 100 total ecosystem carbon for simulations using projected early (2010–19), mid (2050–59), and late (2090–99) century climate with wildfire and historical (1909–2013) climate with wildfire. Mean separation using Tukey’s HSD and 15 replicate simulations of each scenario. (PDF) [file pone.0169275.s014.pdf]

S4 Table: Comparison of year 100 total ecosystem carbon for simulations using projected early (2010-19), mid (2050-59), and late (2090-99) century climate with wildfire and historical (1909-2013) climate with wildfire. Mean separation using Tukey's HSD and 15 replicate simulations of each scenario.

Climate Scenario	Difference	Lower 95% CI	Upper 95% CI	P adj
Historic-Early	26.398948	18.726955	34.070941	<0.0001
Late-Early	-5.215384	-12.887377	2.456609	0.2841514
Mid-Early	-2.223961	-9.895954	5.448032	0.8686933
Late-Historic	-31.614331	-39.286324	-23.942338	<0.0001
Mid-Historic	-28.6229.8	-36.294901	-20.950915	<0.0001
Mid-Late	2.991423	-4.680570	10.663416	0.7312369
