# Supplementary material for: Quantifying the Carbon Balance of Forest Restoration and Wildfire under Projected Climate in the Fire-Prone Southwestern US
Source: PLoS One. 2017 Jan 3;12(1):e0169275. doi: 10.1371/journal.pone.0169275 (PMC5207529; doi:10.1371/journal.pone.0169275)
Supplement: S5 Table — T-test comparison of year 100 total ecosystem carbon for simulations using projected early (2010–19), mid (2050–59), and late (2090–99) century climate with wildfire for seedling establishment probabilities of 0.5 and 1.0. (PDF) [file pone.0169275.s015.pdf]

S5 Table: T-test comparison of year 100 total ecosystem carbon for simulations using projected early (2010-19), mid (2050-59), and late (2090-99) century climate with wildfire for seedling establishment probabilities of 0.5 and 1.0.

Climate Scenario	Pest0.5 ($MgCha^{-1}$)	Pest1.0($MgCha^{-1}$)	p-value
Early	84.8	96.4	0.0007322
Mid	88.5	94.1	0.1365
Late	83.5	91.1	0.02404
